# Impact of Prescribed and Self-Selected Music Interventions on Stress, Sleep, Heart Rate Variability, and Brain Connectivity in Surgeons Using 7-Tesla Functional Magnetic Resonance Imaging and Wearable Actigraphy: Multimodal Feasibility Randomized Controlled Trial

**DOI:** 10.2196/84899

**Published:** 2026-04-17

**Authors:** Mei Rui, Anthony Brandt, Linda Moore, Enshuo Hsu, Christof Karmonik, Aparajitha Verma, Miguel Valdivia y Alvarado, Jefferson Todd Frazier, Atiya Dhala

**Affiliations:** 1 Department of Neurosurgery The University of Texas MD Anderson Cancer Center Houston, TX United States; 2 The Shepherd School of Music Rice University Houston, TX United States; 3 Department of Surgery Houston Methodist Hospital Houston, TX United States; 4 University of Texas MD Anderson Cancer Center Houston, TX United States; 5 Department of Radiology Houston Methodist Hospital Houston, TX United States; 6 University of Texas Health Sciences Houston, TX United States; 7 Center for Performance Arts Medicine Houston Methodist Hospital Houston, TX United States

**Keywords:** actigraphy, burnout, psychological, heart rate, intensive care units, magnetic resonance imaging, music therapy, pilot projects, sleep quality, surgeons

## Abstract

**Background:**

Stress, sleep deprivation, and burnout are significant safety risks for acute care surgeons, negatively impacting performance, well-being, and clinical outcomes.

**Objective:**

This pilot randomized controlled trial aimed to measure neurophysiological effects of prescribed music (PM) and self-selected music (SSM) on surgeon stress, burnout, and neurophysiological responses using a multimodal protocol that integrated functional magnetic resonance imaging (fMRI), wearable biosensor monitoring, and psychological self-assessments.

**Methods:**

Full-time attending surgeons at a quaternary care hospital were invited to participate in a 3-armed trial (1:1:1 block allocation). Intervention groups were instructed to listen to 30 minutes (minimum 15 minutes) of either PM or SSM daily at bedtime for 6 weeks, reflecting real-world conditions. PM comprised original compositions based on elements promoting perceived relaxation from a prior study. The control arm avoided music in the 30 minutes before bed. Allocation was concealed from the recruiting investigator; the fMRI technicians, the statistician, and lead investigators were blinded until analyses were completed. Functional connectivity patterns were measured using fMRI at baseline and 6 weeks while participants listened to simulated intensive care unit noise, PM, and SSM. Secondary outcomes included continuous actigraphy for sleep quality and self-reported anxiety, sleep quality, and burnout using validated scales (State-Trait Anxiety Inventory, Pittsburgh Sleep Quality Index, and Maslach Burnout Inventory).

**Results:**

A total of 22 surgeons were assessed; demands of fMRI and data collection schedule led 3 to decline and 2 (allocated to PM) not to finish baseline measures; 6 PM, 5 SSM, and 6 controls received allocated intervention; 2 PM participants were withdrawn for nonadherence and missing follow-up data and 1 control missed follow-up collection due to scheduling (final analysis set after missing data: PM: n=4, SSM: n=5, control: n=5). One control participant experienced transient vertigo in fMRI. Trends in fMRI data indicated that both intervention groups experienced less negative emotional arousal and anxiety, with physical tension reduced in the PM group. The PM group exhibited reduced stress response in the frontal lobes when exposed to intensive care unit alarms, suggesting diminished attentional response to the high-stress auditory environment, compared to control. However, lack of statistical significance and baseline variability entail cautious interpretation. Observations of sleep quality were mixed, and no statistically significant differences in stress surveys were observed.

**Conclusions:**

Both music interventions trended toward positive changes in neurophysiological responses, suggesting potential benefits in reducing surgeon stress. However, due to the small sample, mixed or nonsignificant results, and the exploratory nature of this study, findings should be considered preliminary. Further research with larger, diverse cohorts is required to confirm trends, refine both the intervention approach and recruitment strategies, and determine whether objective compositional elements or personally selected music drive the mechanisms of potential positive effects.

**Trial Registration:**

ClinicalTrials.gov NCT05980429; https://clinicaltrials.gov/study/NCT05980429

## Introduction

For health care providers working in high-stress, high-autonomy, high-immediacy, and high-risk work environments, stress and sleep deprivation are serious safety hazards. A reported 65% of acute care and subspecialty surgeons experience acute and chronic sleep deprivation [1], and 50%-70% of emergency room physicians report symptoms of burnout [2-4]. Sleep-deprived surgeons are rated to perform with less competence and speed than even their intoxicated peers [5,6]. Numerous studies have emphasized that health care professionals in the intensive care unit (ICU) are highly cognizant of the elevated noise levels, which impair their well-being, performance, and attention [7,8].

Sleep deprivation has also been associated with impaired emotional regulation. Functional magnetic resonance imaging (fMRI) studies have demonstrated that sleep deprivation hinders the ability to down-regulate negative emotions, as evidenced by decreased functional connectivity (FC) between brain regions such as the anterior cingulate cortex and the amygdala, as well as between the frontal lobe and emotional regulation areas [9-14]. Moreover, individuals experiencing high occupational stress have shown reduced brain volumes and diminished FC in areas linked to empathetic responses [15]. Collectively, these findings highlight the vulnerability of prefrontal-limbic regulatory networks in chronically stressed clinical populations [16,17].

Pharmaceutical treatments have traditionally been the main approach for addressing stress- and anxiety-related sleep deprivation. However, these interventions may lose effectiveness over time and carry the risk of addiction [18]. As a promising alternative, music has been suggested as a nonpharmacological, low-risk, and cost-effective intervention to mitigate stress and improve well-being [19].

Music is a well-known modulator of the human stress response [20,21] and has been shown to increase heart rate variability (HRV) [22-24]. HRV, which measures the variation between heartbeats, is an established marker of how the cardiovascular system responds to stress and has served as a biological indicator of overall well-being [25-27]. Higher HRV is generally associated with improved emotional regulation and cardiovascular health, reflecting a more adaptive balance between sympathetic and parasympathetic activity [28-31]. The neurovisceral integration is a functional network linking the prefrontal cortex and the autonomic nervous system, which governs emotional regulation, cognition, and health. Within this framework, higher HRV is associated with stronger prefrontal inhibitory control over subcortical regions such as the amygdala, enhancing cognitive flexibility and adaptive stress responses. Moreover, autonomic regulation manifested by fluctuations in HRV provides a mechanistic link between central neural activity and peripheral physiological responses [32,33].

Beyond its autonomic effects, music has demonstrated the ability to reduce the need for sedatives in clinical populations, alleviate stress, and enhance sleep quality [21]. Music processing engages distributed neural circuits involved in emotion, reward, and empathy, including limbic, paralimbic, and prefrontal regions [34,35], further suggesting its potential therapeutic effects. Neuroimaging studies have shown that pleasurable music listening can engage dopaminergic reward pathways, including the ventral striatum and nucleus accumbens, supporting its role in affective modulation [36]. Recent fMRI studies have further demonstrated that music can enhance FC by promoting integration across cognitive, auditory, motor, and visual systems [37-40]. However, despite this growing literature, there remains limited research directly examining how specific musical interventions differentially modulate FC through identifiable neurophysiological mechanisms, particularly in high-stress professional populations such as surgeons [41]. As Chen et al [41] observed, “If we are ever to harness music’s multitude of influences, we need a solid understanding of the neural circuitry involved and rigorous evidence about how it is engaged by music.”

In our prior research [42], we identified specific features in musical works, such as soft dynamics, minimal accentuation, smooth articulation, and flexible rather than highly pulsed rhythm, that were most effective in enhancing perceived relaxation. Familiar music was also consistently rated as more calming. This evidence-based framework defined compositional elements of relaxation (CERs) that align with resting heart rate (HR) to encourage relaxation. However, this prior work relied on subjective assessments and did not incorporate neuroimaging or objective autonomic measures to evaluate underlying mechanisms.

The purpose of this study was to explore the feasibility of assessing the neurobiological impacts of defined musical interventions on surgeons using multimodal outcome measures. Specifically, we examined whether prescribed music (PM), standardized according to relaxation-promoting compositional features, and self-selected music (SSM), which may engage greater personal relevance, familiarity, and perceived control, would differentially influence FC and physiological stress markers [43]. By integrating neuroimaging, wearable actigraphy, and psychological self-assessments, this pilot study aimed to generate preliminary data to inform future hypothesis-driven investigations.

To accomplish this, the study used a novel methodology incorporating both PM and SSM alongside advanced neuroimaging and wearable technologies. The substantial resource requirements (including access to 7-Tesla fMRI, as detailed in the Methods section) and the scheduling constraints inherent to a surgical population warranted an initial pilot design.

## Methods

### Trial Design, Participants, and Recruitment

This pilot study was conducted at a quaternary care hospital from July 2020 to June 2021 to evaluate the feasibility of assessing the impact of music interventions on surgeon stress, burnout, and neurophysiological responses. The parallel randomized controlled design (NCT05980429) involved a small sample of full-time attending surgeons in acute care or other surgical subspecialties. Inclusion criteria required participants to be actively practicing surgeons, while those diagnosed with sleep apnea, hearing and cognitive impairments, metal implants, or self-reported regular use of prescribed sleep medications were excluded.

Between June 2020 and November 2021, potential participants were identified by the principal investigator (PI) from the Department of Surgery at Houston Methodist Hospital based on inclusion criteria. Eligible participants received study invitations via email, which included an overview of the research objectives, procedures, and contact details of the research team for further inquiries. The PI subsequently approached interested individuals in person for a detailed discussion about the study and to obtain written informed consent. Cognizant of the gender imbalance in the department (few women), the PI reached out to eligible specialist surgeons in urology and vascular to obtain a more representative sample within the predefined inclusion criteria.

Randomization followed a computer-generated 1:1:1 block design using Stata (version 16; Stata Corp LLC) for number generation. Demographic characteristics, such as age, gender, race, and surgical specialty, were recorded for each participant. Baseline assessments were conducted in week 1, followed by the application of the music intervention or control from week 2 to week 6. End-of-study assessments were completed on the final day of week 6.

### Intervention

The PM group was instructed to listen to PM (recommended 30 minutes, minimum 15 minutes) at bedtime. The SSM group was asked to listen to 30 minutes of music of their choosing at bedtime. The control group was instructed not to listen to music during the 30-minute period before bedtime preparation period. Blinding participants to their groups was not possible due to the nature of the intervention; however, allocation was concealed from the recruiting investigator, and the fMRI technicians, the statistician, and all lead investigators were blinded to participants' groups until all analyses were completed.

The musical works selected for the prescribed intervention contained CERs (Textbox 1), identified and evaluated for their impact on promoting perceived relaxation in a demographically diverse cohort [42,44]. The selected pieces were analyzed, prepared, and recorded by professional musicians (samples available [44]). Music used for the study was downloaded from a password-protected Google Drive accessible to the study participants assigned only to the PM group. The SSM consisted of participants’ own musical tracks without exclusions and did not overlap with the PM.

Participants were instructed to wear noise-canceling headphones to reduce distractions from external stimuli and were asked to note whether they fell asleep while listening to music. Each participant reported their adherence to the music assignment on a weekly basis using a 5-question survey (tabulated below in Results).

**Compositional elements of relaxation used to select prescribed music.**

**Inclusion criteria**

- Without strong accents
- Legato and portamento articulation
- Pianissimo to mezzo-forte range
- Unfamiliar
- Master performer
- Smooth melodic shape
- High recording quality
- None to minimal repetition
- Low to middle frequency 50-1000 Hz; (eg, cello, piano, viola, horn, and harp)
- Rubato with flexibility
- Tempo of 60-90 beats per minute
- Lower/middle strings, piano, lower/middle-range winds, and harp
- Time period from 1650 to modern
- Tonality characterized by major and minor keys, pentatonic, and consonances

**Exclusion criteria**

- With strong accents
- Staccato articulation
- Forte to fortissimo range
- Familiar
- Amateur/mediocre performer
- Jagged melodic shape with large leaps
- Low to medium recording quality
- Excessive repetition
- High frequency >1000 Hz (eg, piccolo and upper strings)
- Rigid rubato, without flexibility
- Tempo above 95 beats per minute
- Vocal music, upper strings, and high-frequency winds (eg, piccolo)
- Pre-1650 time period
- Atonal, unpredictable melodic and/or harmonic structures, and excessive dissonances

### Outcome Measures

#### FC and fMRI

The primary mechanistic outcome tested in this pilot and to be proposed at scale was the change in brain FC. The 7-Tesla fMRI (Siemens MAGNETOM Terra [Siemens Healthineers]) was used to measure this outcome. To replicate the high-stress auditory environment routinely experienced by acute-care surgeons and intensivists, all participants were exposed to ICU noise during the fMRI sessions. We selected ICU noise as the control stimulus rather than a “neutral” baseline of ambient hospital noise to represent a more ecologically valid working environment. In critical care, periods of peak concentration and high-stakes decision-making occur at periods of substantial and meaningful background noise. In contrast to clinicians in, for instance, the operating room, who can regulate their background conditions (including the option to use music), the ICU soundscape is largely uncontrollable and can be persistently intrusive; therefore, ICU noise provides a realistic reference condition against which to evaluate potential effects of both PM and SSM.

All groups listened to the PM playlist, specifically curated to include CERs aimed at promoting relaxation, as well as SSM based on individual preferences. The ICU noise, PM, and SSM were played in the same sequence for all participants during the baseline and 6-week follow-up fMRI sessions.

To quantify the strength of FC, we used the Analysis of Functional NeuroImages (AFNI) software (National Institute of Mental Health, Scientific and Statistical Computing Core), which allowed for a more detailed quantitative calculation of FC. Following standard AFNI preprocessing, the acquired fMRI images were spatially normalized into Talairach space using the TT_icbm452 atlas as the reference. Each participant’s brain was parcellated into anatomically defined regions based on this atlas. The analysis focused on detecting changes between brain regions associated with empathetic responses (anterior cingulate cortex, a region linked to empathy and compassionate behavior, and one where oxytocin receptors are expressed) [45,46] and pain perception and multisensory awareness (insula region) [47]. For more details, see Multimedia Appendix 1.

The fMRI scans were performed at baseline (week 1) and again at the end of the study (week 6). Harms, such as transient vertigo, were monitored during the fMRI sessions. This methodology was selected to test the feasibility of using advanced neuroimaging techniques to evaluate the neurophysiological impact of music interventions in this population of surgeons. The ICU noise, PM, and SSM were played in the same sequence for all participants during the baseline and 6-week follow-up fMRI sessions.

To quantify the strength of connectivity within defined brain networks, graph structures were constructed using the *igraph* package in the R software (version 4.3.1; R Foundation for Statistical Computing), where each node represented a brain region and the edge weights were defined by the Pearson correlation coefficients between Blood Oxygen Level Dependent time series. For each network, the average of the edge weights between all region pairs belonging to that network was used as a summary measure of network connectivity strength.

#### Secondary Outcomes

##### Wearable 3-Axis Actigraphy and Sleep Activity

The WHOOP (version 3.0; Whoop, Inc) wearable actigraphy wristband was used to assess the selected secondary outcomes: resting HR, HRV, and sleep activity. The WHOOP records 100 measurements per second, each of HR, HRV, ambient temperature, skin conductivity, and movement via a 3-axis accelerometer. It can detect rapid eye movement (REM) sleep, 2 stages of light and slow-wave sleep (SWS), and the time to sleep (latency). The device’s accuracy (86%), sensitivity (90%), and positive predictive value (93%) are well established [48]. WHOOP data were accessed through the WHOOP application programming interface.

Participants were asked to wear the WHOOP device continuously over a 6-week period and download the WHOOP smartphone app to create a deidentified profile. Daily and average weekly WHOOP sleep scores were recorded. All participants were required to log into the WHOOP app on their phones daily to ensure uninterrupted data collection during on-call and noncall nights. The app also recorded data on daytime naps. The number of hours worked and days on call per week were also recorded to assess potential confounding factors related to sleep patterns. No standardized restrictions were imposed regarding hydration status, caffeine and alcohol intake, or physical activity during the 6-week monitoring period. Participants were instructed to follow their usual daily routines.

##### Measures of Stress

Secondary outcomes also included self-reported measures of anxiety, sleep quality, and burnout. Stress was assessed using the State-Trait Anxiety Inventory [49], a validated tool for measuring anxiety levels, with lower scores reflecting less anxiety. The Pittsburgh Sleep Quality Index [50] was used to assess sleep quality, with lower scores indicating better perceived sleep. To measure burnout, the Maslach Burnout Inventory [49] was administered, focusing on levels of occupational stress. All surveys were administered at baseline (week 1) and again at the end of the study (week 6) to assess potential changes over the course of the intervention.

### Statistical Analysis

Participant characteristics and baseline sleep measurements were reported with summary statistics, stratified by intervention groups. Power calculations were not conducted at this pilot stage; rather, the number of participants assessed for eligibility (see the Results section) was based on initial interest in response to the email invitation.

To evaluate the impact of music intervention on sleep measurements, for each intervention group and each sleep measurement, a generalized estimating equations (GEE) model was applied to model the sleep outcomes of participants over time with music intervention as a predictor, adjusted on previous sleep debt, naps, and music-listening compliance. First-order autoregressive covariate structure and a Gaussian distribution were used in the GEE model to handle the multiple measurements of each participant over time.

To investigate the impact of music intervention, logistic regression was applied using music intervention as the predictor, adjusted for previous sleep debt and naps. The preintervention and postintervention surveys were summarized using mean ± standard deviation and compared using a paired t-test. Participants who had inconsistent music listening adherence were dropped from the GEE analysis and logistic regression analysis.

Specifically, for the fMRI data interpretation, changes in brain network connectivity strength were calculated by comparing postintervention connectivity values to those obtained at baseline. This comparison was conducted separately for each participant and each network. By aggregating across participants within experimental groups, it was possible to identify patterns of increased or decreased FC across the somatomotor, limbic, stress-related, and other canonical brain networks. These longitudinal changes provided insight into the neurofunctional effects of the intervention and were statistically evaluated using paired 2-tailed *t* tests.

Analyses were performed using Stata version 16 with significance set at *P*<.05. Given the exploratory nature of this study, these analyses are intended to provide preliminary insights and inform the design of future, larger-scale studies.

### Ethical Considerations

The study protocol was approved by the Houston Methodist Research Institute Institutional Review Board (protocol number Pro00024163). All participants provided informed consent. Participants did not receive financial compensation or incentives for their involvement in the study. No identifiable data are presented in this report.

## Results

### Overview and Participant Demographics

**Figure 1:**
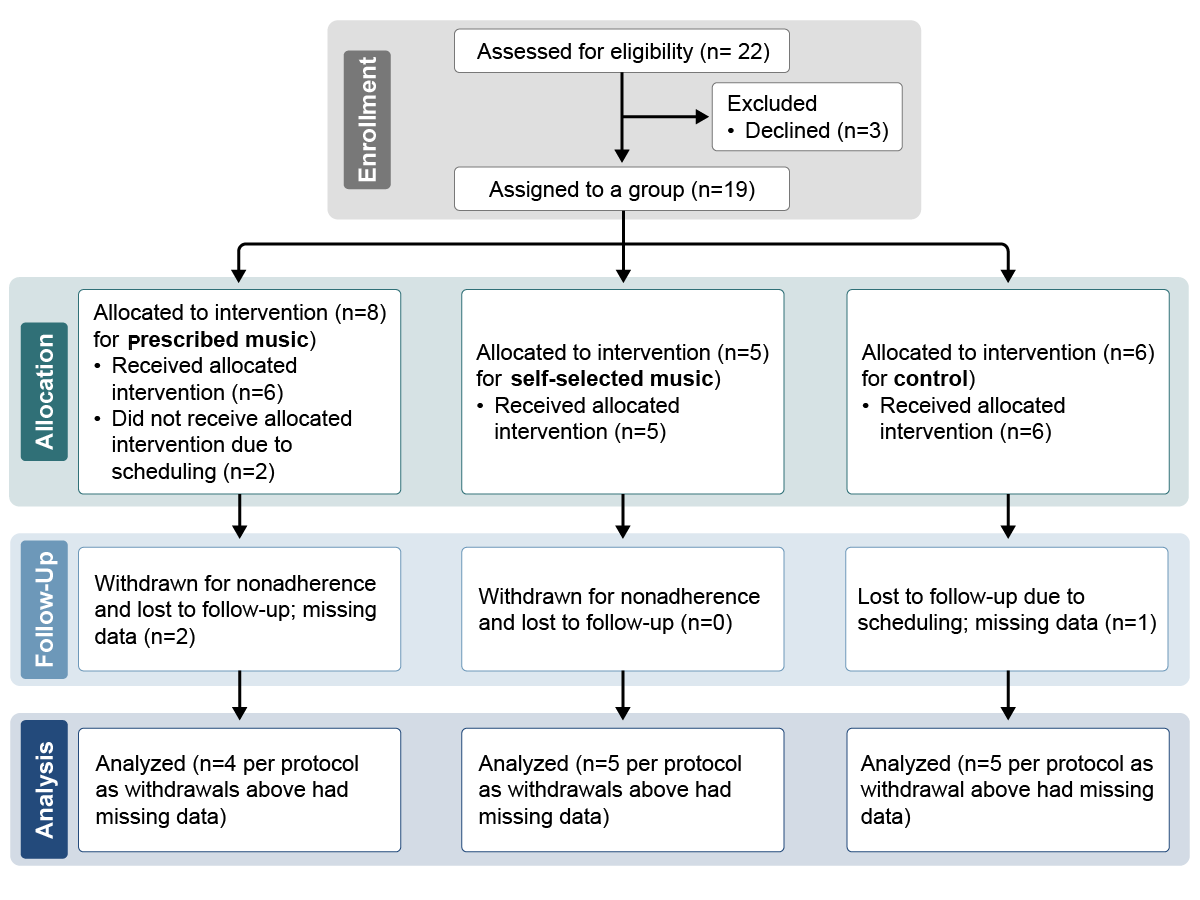
Participant flow diagram.

A total of 22 surgeons expressed interest in the study, though scheduling difficulties led to 3 declinations and additional withdrawals with missing follow-up data (Figure 1). In the PM group, 2 participants had challenges with adherence and were unable to complete the study (reported inconsistent music listening and WHOOP data, with no follow-up fMRI data collected); only 4 participants were included in the analyses for this group. The SSM and control groups each included 5 participants, after excluding 1 control participant who did not complete follow-up data collection for analysis.

The average participant age was 45 (SD 9) years, with most participants identifying as non-Hispanic White (9/14, 64.3%) and male (13/14, 92.9%). Most participants (9/14, 64.3%) had a subspecialty other than acute care surgery (Table 1). No harms were reported from music listening or headphone use. One participant in the control group experienced vertigo in the fMRI, which was resolved by a brief period of rest. Adherence to the intervention, notwithstanding the withdrawals due to scheduling, was acceptable among the remaining 4 who filled the adherence survey (Table 2).

The SSM group chose a heterogeneous mixture of tracks: “Erik Satie’s Gymnopédie” for piano and “Beethoven’s Symphony No. 9,” both classical works; rock works “Bohemian Rhapsody” (Queen) and “Sur L’Ocean Couler de Fer” (Alcest); and the alternative trip hop track “Aqualung” (Morcheeba). To assist in interpreting the effect of variability within the self-selected tracks and points of convergence or divergence from attributes of CER, the authors classified these tracks (Multimedia Appendix 2).

**Table 1:** Baseline demographic characteristics of general and acute surgeons participating in the music intervention sleep study.

Characteristic			Overall (n=14)		Prescribed music group (n=4)		Self-selected music group (n=5)		Control group (n=5)		*P* value^a^	
Age (years), mean (SD)			45.2 (9.0)		46.2 (12.1)		45.8 (11.1)		43.8 (5.4)		.92	
**Gender, n (%)**												.38
	Female	1 (7.1)		0 (0)		0 (0)		1 (20)				
	Male	13 (92.9)		4 (100)		5 (100)		4 (80)				
**Race and ethnicity, n (%)**												.47
	Asian	3 (21.4)		0 (0)		1 (20)		2 (40)				
	Hispanic	2 (14.3)		1 (25)		0 (0)		1 (20)				
	Non-Hispanic White	9 (64.3)		3 (75)		4 (80)		2 (40)				
**Specialty, n (%)**												.63
	Surgical subspecialty	9 (64.3)		2 (50)^b^		4 (80)^c^		3 (60)^d^				
	Acute care surgery	5 (35.7)		2 (50)		1 (20)		2 (40)				

^a^For numeric variables, the ANOVA test was used; for categorical variables, the chi-square test was used.

^b^General surgery (oncology) and transplant surgery.

^c^Colorectal surgery, general surgery (bariatric), general surgery (oncology), and thoracic surgery.

^d^General surgery (bariatric), thoracic surgery, and transplant surgery.

**Table 2:** Participant self-reported adherence to study protocol for those not lost to follow-up^a^.

Question/response		PM^b^, n (%)	SSM^c^, n (%)	*P* value^d^	
**Question 1^e^**					.52
	4-5	2 (50)	4 (80)		
	6-7	2 (50)	1 (20)		
**Question 2^f^**					.49
	10-20	1 (25)	2 (40)		
	20-30	3 (75)	2 (40)		
	30-45	0	1 (20)		
**Question 3^g^**					>.99
	50-74	0	1 (20)		
	75-100	4 (100)	4 (80)		
**Question 4^h^**					>.99
	Yes	4 (100)	5 (100)		
	No	0	0		
**Question 5^i^**					.36
	2	0	1		
	4	0	1		
	5	1	0		
	6	0	2		
	7	1	0		
	8	2	0		
	9	0	1		

^a^Early withdrawals due to scheduling, inconsistent listening throughout the study, or wearing the WHOOP device <50% of the time while listening to music, precluded full analysis due to missing data (ad hoc determination by study statistician).

^b^PM: prescribed music.

^c^SSM: self-selected music.

^d^Paired *t* tests were conducted for *P* values.

^e^Question 1: How frequently were you able to follow the listening protocol, nights per week? (Count).

^f^Question 2: Approximately how long did you listen to music each time? (Minutes).

^g^Question 3: While listening to the music, what percentage of the time were you wearing the WHOOP device? (Percent).

^h^Question 4: During your on-call days/nights, did you wear the WHOOP device?

^i^Question 5: On a scale of 1-10, how would you characterize your work stress level during the study period? (1=minimal level of stress and 10= maximum level of stress; table shows only scores selected by at least 1 participant).

### Findings From fMRI

**Figure 2:**
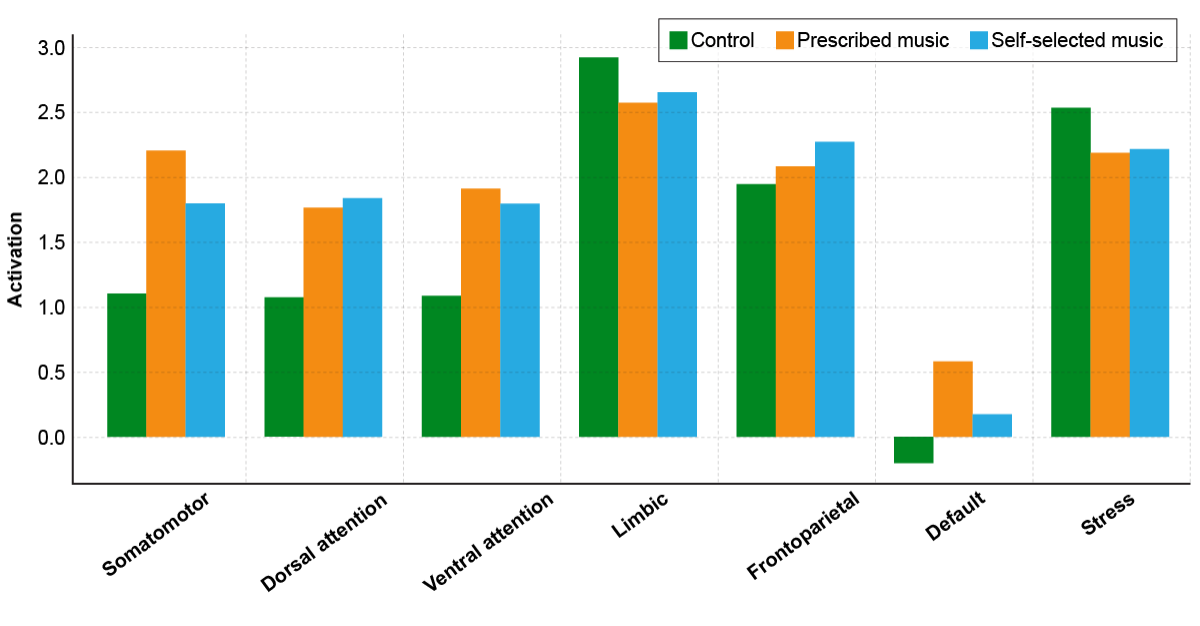
Baseline activation of measured brain regions across groups.

At baseline (preintervention), after exposure to ICU noise, PM and SSM were played in a randomized sequence for each participant. Therefore, the observed differences in brain network activation across the 3 participant groups reflect inherent variability in individual functional brain connectivity related to these 3 audio stimuli (Figure 2 and Table 3). Some networks, such as the limbic and stress-related systems, show notably higher mean activation in the control and PM groups, suggesting natural differences in emotional and physiological regulation. The somatomotor and frontoparietal networks also exhibit variability that may relate to differences in intrinsic motor control and cognitive engagement among participants. These baseline values provide an important reference point for evaluating the effects of the intervention and help establish individual and group-level differences in neural network dynamics under noninterventional conditions.

**Table 3:** Brain network connectivity strength at baseline.

Network	Control, mean (SD)	Prescribed music, mean (SD)	Self-selected music, mean (SD)
Somatomotor	1.11 (3.26)	2.21 (2.56)	1.81 (3.27)
Dorsal attention	1.08 (2.82)	1.78 (2.25)	1.85 (2.11)
Ventral attention	1.09 (2.17)	1.93 (1.26)	1.80 (1.69)
Limbic	2.94 (1.42)	2.59 (0.63)	2.67 (1.01)
Frontoparietal	1.95 (1.41)	2.10 (1.33)	2.29 (1.84)
Default	–0.20 (1.80)	0.59 (1.95)	0.18 (1.95)
Stress	2.54 (1.17)	2.20 (0.85)	2.23 (1.05)

**Figure 3:**
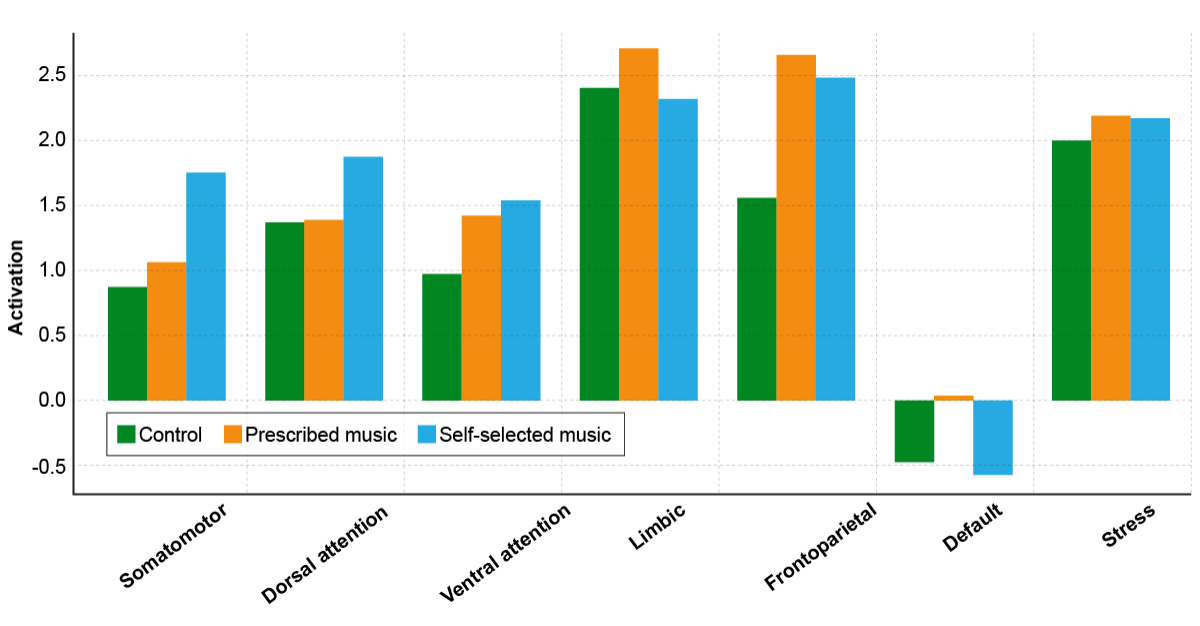
Postintervention activation of measured brain regions across treatment groups.

At the 6-week follow-up fMRI (postintervention), ICU noise, PM, and SSM were played in the same sequence for all participants as during the preintervention scan. Following the music listening during the 6-week follow-up fMRI scan (postintervention), most brain networks showed a general reduction in activation across all groups (Figure 3 and Table 4). This was especially notable in networks such as the somatomotor and default mode networks. The consistent decline suggests a calming or regulatory effect of music exposure during the scan, potentially reducing motor readiness and default internal thought processes across the board.

**Table 4:** Brain network connectivity strength measured postintervention.

Network	Control, mean (SD)	Prescribed music, mean (SD)	Self-selected music, mean (SD)
Somatomotor	0.88 (3.34)	1.08 (3.78)	1.76 (3.52)
Dorsal attention	1.38 (2.52)	1.39 (2.11)	1.88 (2.15)
Ventral attention	0.97 (1.95)	1.43 (1.68)	1.54 (1.78)
Limbic	2.40 (1.05)	2.70 (1.16)	2.32 (0.92)
Frontoparietal	1.56 (2.02)	2.66 (2.30)	2.49 (1.93)
Default	–0.47 (1.32)	0.04 (1.75)	–0.57 (2.01)
Stress	1.99 (1.24)	2.19 (1.22)	2.17 (1.08)

**Figure 4:**
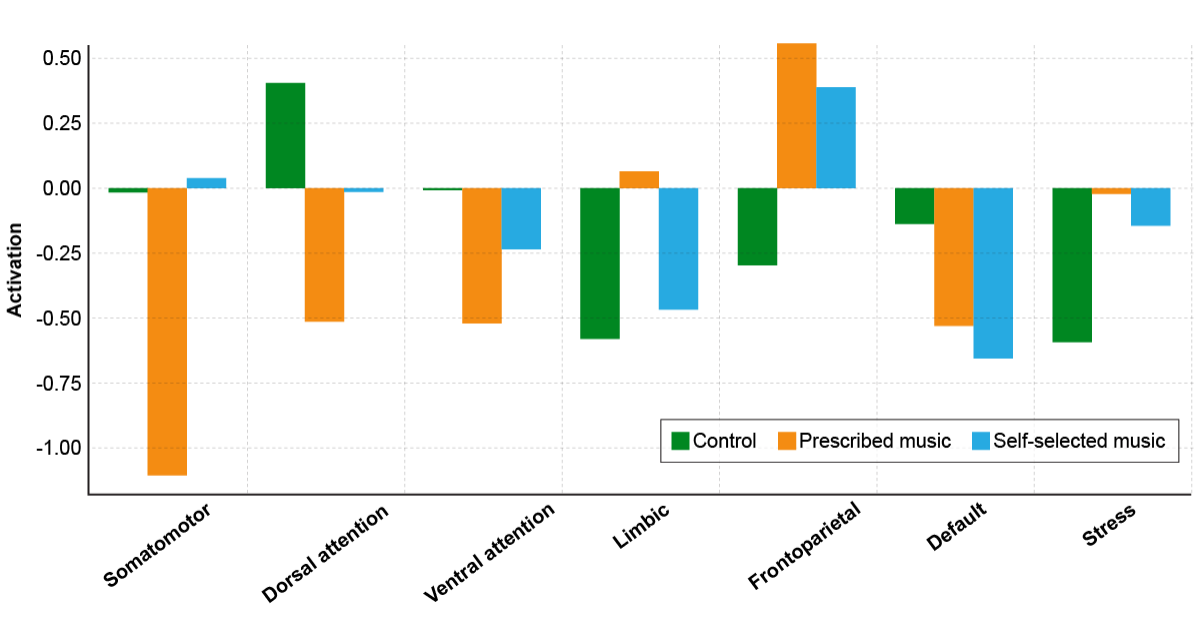
Difference in activation of measured brain regions (from baseline to postintervention) across treatment groups.

Differences between the follow-up and baseline conditions did not reach statistical significance within the context of this pilot-sized study, though trends are noted here to indicate how the intervention may influence each brain network (Figure 4 and Table 5). The somatomotor network, responsible for motor control and bodily movement, showed a marked reduction in the PM group, suggesting a potential decrease in areas associated with physical tension. The limbic system, central to emotion processing and memory, showed a reduction, albeit not statistically significant, in the SSM group, which could reflect emotional calming or reduced anxiety levels. Although the stress network exhibited a decline in the control group in terms of effect size, signifying reduced stress or arousal at follow-up, we could not establish statistical significance.

**Figure 5:**
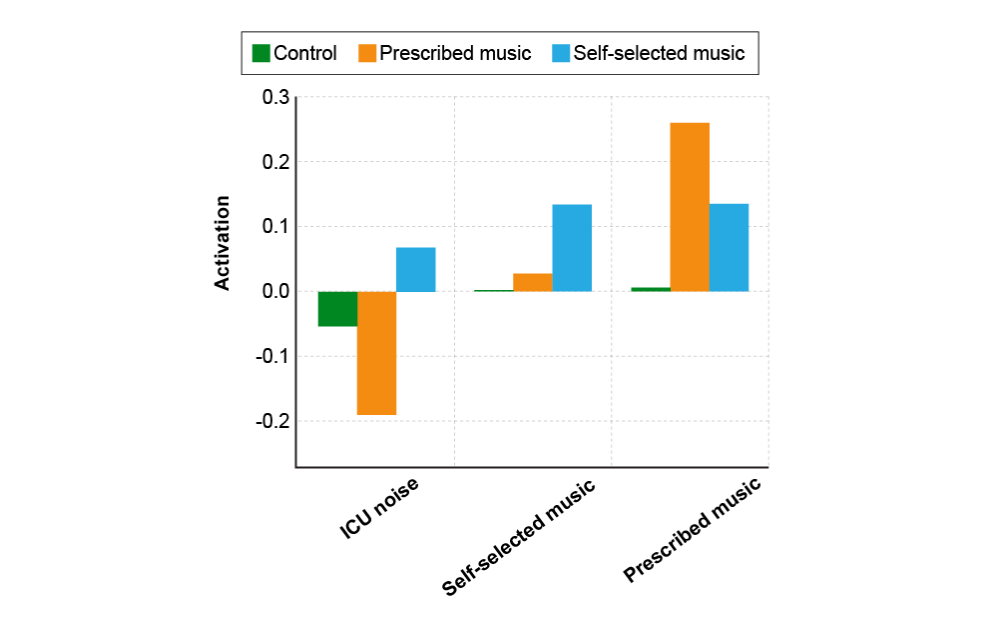
Changes in total brain connectivity after music listening intervention relative to baseline. ICU: intensive care unit.

Nonsignificant differences were also observed in how each group responded to stress-triggering ambient ICU noise during the fMRI scan (Figure 5). Both the PM and SSM groups showed large changes in FC, including both strengthening and dissociation of FC in disparate brain regions. In particular, the PM and SSM groups exhibited overlapping results: both groups showed increased FC in the cerebellar areas and inferior parietal lobes—regions associated with empathy, movement planning and coordination, decision-making, spatial attention, and body-sensory awareness. Notably, when exposed to stress-triggering ICU alarms, the PM group exhibited lower FC in the frontal lobes and hippocampus—regions involved in attention and physiological regulation—compared to control and SSM. Additionally, the PM group showed strengthened FC in the occipital lobes, associated with visual, spatial, and melodic processing and memory encoding, and the parietal regions, associated with attention and sensory integration, suggesting that the intervention may have a shielding effect against negative, stress-inducing environmental acoustic stimuli.

**Table 5:** Difference between brain network connectivity strength (from baseline to postintervention).

Network	Control			Prescribed music			Self to selected music		
	Mean difference (95% CI)	*P* value^a^	Mean difference (95% CI)		*P* value	Mean difference (95% CI)		*P* value	
Somatomotor	–0.02 (–1.85 to 1.81)	.98	–1.11 (–3.35 to 1.13)		.18	0.04 (–1.84 to 1.92)		.95	
Dorsal attention	0.40 (–1.08 to 1.88)	.64	–0.52 (–2.03 to 0.99)		.48	–0.02 (–1.20 to 1.16)		.98	
Ventral attention	–0.01 (–1.15 to 1.13)	.99	–0.52 (–1.55 to 0.51)		.21	–0.24 (–1.20 to 0.72)		.52	
Limbic	–0.58 (–1.27 to 0.11)	.21	0.07 (–0.58 to 0.72)		.84	–0.47 (–1.00 to 0.07)		.16	
Frontoparietal	–0.30 (–1.27 to 0.67)	.53	0.56 (–0.74 to 1.86)		.42	0.39 (–0.66 to 1.44)		.50	
Default	–0.14 (–1.01 to 0.73)	.81	–0.54 (–1.82 to 0.74)		.30	–0.66 (–1.76 to 0.44)		.30	
Stress	–0.60 (–1.27 to 0.07)	.19	–0.03 (–0.76 to 0.70)		.94	–0.15 (–0.74 to 0.44)		.69	

^a^Longitudinal changes were statistically evaluated using within-group paired *t* tests (α=.05) comparing pre-post intervention for each network; unit of analysis = mean network strength per participant.

The SSM group exhibited a decrease in FC in the default mode network, potentially reflecting a shift in focus toward processing personal memories related to the SSM music. Contrary to PM, the SSM group showed reduced FC in the occipital lobes, potentially due to the familiarity of the SSM music compared to the PM, and an increase in FC in the temporal gyrus, a region involved in memory processing, though these findings would need to be confirmed in larger studies, as trends were not statistically significant. Notably, neither the PM nor the SSM groups demonstrated any decreases in FC in the anterior cingulate or insula regions—key areas for emotional processing and bodily awareness—suggesting that the music interventions provided a shielding, protective effect against the environmental acoustic stressors (ICU alarms).

The control group showed small changes in FC post intervention compared to baseline. FC in the executive regions increased, suggesting heightened awareness of the ICU noise. Decreases in FC were observed in the anterior cingulate and insula regions, indicating that the ICU noise may have a suppressive effect on emotional regulation and bodily awareness. These changes may reflect a negative neural response to the stressful environment of the ICU (again, to be confirmed to ensure significant results).

### Findings From WHOOP

Overall, the baseline sleep latency was 127.8 seconds, time in bed was 6.8 hours per sleep, with SWS duration of 1.4 hours, and REM sleep duration of 1.7 hours (Table 6). Participants had resting HR of 60.0 and HRV of 49.1 milliseconds (root-mean-square of successive differences between normal heartbeats). No significant differences in sleep and HR were observed between the groups at baseline.

Participants in the PM group experienced no change in sleep activity during the study (Table 7). After 6 weeks of SSM intervention, participants in this group were in bed longer (0.49 hours; *P*=.01), slept longer (0.39 hours; *P*=.01), and spent more time in light sleep (0.3 hours; *P*<.001). Overall, the SSM group experienced a 0.84% decrease in sleep efficiency (*P*=.001). The control group had no significant changes in sleep activity.

In the PM group, observations were mixed: 1 participant had increased HRV by 9.74 milliseconds while another participant decreased by 11.37 milliseconds, and in 2 participants, no change was noted (Table 8). In the SSM group, the individual logistic regression showed an increased HRV post intervention, ranging from 1.31 to 11.55 milliseconds. This result is consistent with the GEE model, which estimates an overall increase of 2.76 milliseconds (*P*=.03). The control group showed no change in HRV from baseline to the end of the study.

**Table 6:** Baseline sleep characteristics of acute and subspecialty surgeons^a^.

Metric			Overall		Prescribed music group		Self-selected music group		Control group		*P* value
**Baseline sleep activity**											
	Latency (seconds), mean (SD)	127.8 (74.4)		142.4 (148.5)		132.2 (29.2)		111.7 (4.4)		.84	
	Time in bed (hours), mean (SD)	6.8 (1.0)		7.3 (0.3)		6.5 (1.0)		6.6 (1.4)		.60	
	Light sleep duration (hours), mean (SD)	3.0 (0.6)		3.1 (0.5)		3.0 (0.6)		2.9 (0.8)		.83	
	Slow-wave sleep duration (hours), mean (SD)	1.4 (0.2)		1.4 (0.2)		1.3 (0.1)		1.4 (0.2)		.53	
	REM^b^ sleep duration (hours), mean (SD)	1.7 (0.4)		2.1 (0.2)		1.5 (0.4)		1.7 (0.2)		.07	
	Total sleep duration (hours), mean (SD)	6.1 (0.9)		6.6 (0.5)		5.9 (0.9)		6.0 (1.2)		.54	
	Wake duration (hours), mean (SD)	0.6 (0.3)		0.7 (0.3)		0.6 (0.2)		0.6 (0.3)		.85	
	Arousal time (hours), mean (SD)	0.2 (0.1)		0.2 (0.0)		0.3 (0.1)		0.3 (0.1)		.49	
	In-sleep efficiency (%), mean (SD)	91.6 (2.9)		91.6 (3.4)		91.5 (2.0)		91.8 (3.9)		.98	
	Respiratory rate (breaths/minute), mean (SD)	15.5 (1.1)		15.5 (1.0)		15.4 (0.6)		15.6 (1.6)		.98	
	Disturbances count, median (IQR)	5.9 (4.6-8.3)		5.6 (4.7-6.40)		7.0 (4.6-8.7)		5.6 (4.7-9.3)		.70	
**Baseline recovery, mean (SD)**											
	Resting heart rate (bpm)	60.3 (11.3)		64.4 (15.8)		62.5 (9.5)		54.9 (8.8)		.43	
	HRV^c^ rMSSD^d^ (ms)	49.1 (21.2)		59.5 (26.7)		37.5 (20.4)		52.3 (14.8)		.30	

^a^For continuous variables summarized with mean (SD), the ANOVA test was used; for ordinal variables summarized with median (IQR), the Kruskal-Wallis test was used; for categorical variables, the chi-square test was used.

^b^REM: rapid eye movement.

^c^HRV: heart rate variability.

^d^rMSSD: root-mean-square of successive RR interval differences.

**Table 7:** Generalized estimating equation model comparing the change in sleep activity from pre- to postintervention for all groups.

Variables	Prescribed music group (n=4), coefficient (95% CI)	*P* value^a^	Self-selected music group (n=5), coefficient (95% CI)	*P* value	Control group (n=5), coefficient (95% CI)	*P* value
Δ in sleep latency (seconds)	9.21 (–5.00 to 23.42)	.20	15.62 (–21.71 to 52.95)	.41	49.67 (–7.93 to 107.28)	.09
Δ in sleep efficiency (%)	–2.78 (–5.58 to 0.02)	.05	–0.84 (–1.35 to 0.33)	.001	–1.1 (–4.05 to 1.85)	.47
Δ time in bed (hours)	0.27 (–0.18 to 0.72)	.24	0.49 (0.11 to 0.86)	.01	–0.18 (–0.69 to 0.33)	.49
Δ total sleep duration, (hours)	0.09 (–0.10 to 0.28)	.36	0.39 (0.08 to 0.69)	.01	–0.21 (–0.77 to 0.36)	.47
Δ light sleep duration (hours)	0.00 (–0.29 to 0.30)	.98	0.3 (0.15 to 0.45)	<.001	0.01 (–0.31 to 0.33)	.95
Δ REM^b^ duration (hours)	0.07 (–0.17 to 0.30)	.58	0.07 (–0.11 to 0.26)	.45	–0.10 (–0.23 to 0.03)	.13
Δ slow-wave duration (hours)	0.05 (–0.02 to 0.12)	.19	0.04 (–0.06 to 0.14)	.42	–0.10 (–0.26 to 0.06)	.23

^a^Generalized estimating equation model and Wald test statistic were used to calculate *P* values.

^b^REM: rapid eye movement.

**Table 8:** Generalized estimating equation model comparing the change in heart rate and variability from pre- to postintervention for all groups.

Variables	Prescribed music group (n=4), coefficient (95% CI)	*P* value^a^	Self-selected music group (n=5), coefficient (95% CI)	*P* value	Control group (n=5), coefficient (95% CI)	*P* value
Δ resting heart rate (bpm)	–0.82 (–2.43 to 0.80)	.32	1.31 (–0.43 to 3.05)	.14	1.71 (–3.69 to 7.11)	.53
Δ HRV^b^ rMSSD^c^ (ms)	–2.04 (–8.71 to 4.63)	.55	2.76 (0.35 to 5.16)	.03	4.51 (–5.15 to 14.16)	.36

^a^Generalized estimating equation model and Wald test statistic were used to calculate *P* values.

^b^HRV: heart rate variability.

^c^rMSSD: root-mean-square of successive RR interval differences.

### Subjective Measures of Stress

No statistically significant differences were observed in the self-reporting surveys after music intervention (Table 9).

**Table 9:** Summary of subjective stress survey measures.

Survey component and study period		Prescribed music (n=4)	Self-selected music (n=5)	Control (n=5)
**State anxiety^a^**				
	Pre, mean (SD)	31.25 (6.90)	29.20 (5.07)	30.30 (4.35)
	Post, mean (SD)	27.25 (7.18)	26.20 (3.63)	29.00 (3.08)
	*P* value^b^	.11	.27	.63
**Trait anxiety^a^**				
	Pre, mean (SD)	37.75 (8.45)	35.70 (7.28)	32.30 (5.56)
	Post, mean (SD)	37.00 (9.52)	32.00 (5.66)	34.60 (8.82)
	*P* value	.80	.12	.70
**Global PSQI^c^**				
	Pre, mean (SD)	7.50 (1.00)	4.50 (0.58)	6.00 (3.87)
	Post, mean (SD)	6.50 (2.08)	4.75 (0.96)	6.20 (4.44)
	*P* value	.25	.64	.90
**Burnout** ^d^				
	Pre, mean (SD)	15.75 (11.24)	11.00 (10.61)	8.80 (3.42)
	Post, mean (SD)	8.25 (5.12)	8.20 (6.61)	8.60 (3.51)
	*P* value	.13	.23	.80
**Depersonalization^d^**				
	Pre, mean (SD)	11.00 (10.61)	7.20 (4.60)	8.20 (4.15)
	Post, mean (SD)	8.20 (6.61)	6.60 (4.47)	4.00 (2.00)
	*P* value	.23	.50	.06
**Personal achievement^d^**				
	Pre, mean (SD)	8.80 (3.42)	43.80 (3.83)	40.80 (1.30)
	Post, mean (SD)	8.60 (3.51)	45.00 (3.54)	42.40 (2.41)
	*P* value	.80	.58	.10

^a^From State-Trait Anxiety Inventory; higher scores indicate greater anxiety. Missing answers were filled with 2.5 points.

^b^Paired 2-tailed *t* tests were conducted to compare the pre-post scores.

^c^PSQI: Pittsburgh Sleep Quality Index; higher global scores indicate worse sleep quality.

^d^Maslach Burnout Inventory; for Burnout and Depersonalization, higher scores indicate higher-level burnout; for Personal achievement, higher scores indicate lower-level burnout.

## Discussion

### Principal Findings and Comparisons to Literature

This pilot feasibility study suggested potential associations between music interventions and changes in the neurophysiology of surgeons. While results did not show significant changes across groups, the use of precision tools such as 7-Tesla fMRI for assessing brain FC and 3-axis actigraphy for evaluating sleep and HRV showed promise in detecting subtle differences between participants who received music interventions and those who did not. These precision tools provided valuable insights into the potential impact of music on stress reduction and merit use in larger, confirmatory studies, providing a foundation for future research efforts.

In 2019, Coleman et al [1] published a study using WHOOP actigraphy to examine the prevalence and pattern of sleep deprivation among acute care surgeons. While identifying consistent patterns of acute and chronic sleep deprivation among these surgeons, the study did not test any type of intervention regimen. To our knowledge, ours is the first study that has tested PM and SSM interventions and quantified their effects using fMRI and wearable actigraphy.

Participants in the PM group exhibited reduced FC in the frontal lobes while listening to ICU noise in the 7-Tesla fMRI scanner, suggesting that music may have diminished their negative attentional response to an induced high-stress auditory environment. In contrast, the control group showed an increase in FC in the frontal lobes when exposed to ICU noise, indicating heightened distraction and impaired focus. The control group also had reduced connectivity in brain areas tied to emotion and bodily awareness, suggesting a negative impact of ICU noise on emotional processing.

Compared to baseline, the PM group also showed reduced activity in the somatomotor network, possibly reflecting less physical tension. The SSM group showed a nonsignificant reduction in the limbic system, which could signal emotional calming.

These findings are particularly noteworthy given the well-documented adverse effects of noise pollution in high-stress occupational environments, which include increased cortisol release [51], oxygen consumption, and hypertensive risks, along with impaired sleep quality [52-55]. Our results suggested that regular musical exposure can reduce the negative neural impact on practicing surgeons caused by excessive alarms, beeping, and other ICU acoustic stimuli. While many studies have focused on ICU noise and how it distracts clinicians [7,8], our study went further to assess how music intervention impacts the surgeons’ FC and their ability to concentrate on the critical tasks at hand while blocking out the ICU noise.

Both music interventions showed promising, though nonstatistically significant, fMRI trends, as reflected in the changes in FC found at the end of the study compared with baseline. Despite the small number of participants, our findings indicate that music may modulate the human stress response, but the exact neurophysiological mechanisms underlying these effects require further exploration. These preliminary insights into the impact of music on brain connectivity offer an important starting point for refining the intervention protocols and targeting larger populations in future studies.

Notably, while both the PM and SSM showed neurological changes not found in the control group, their outcomes differed. Choice of repertoire was likely a contributing factor; for instance, the prescribed playlist was controlled and homogenous to include CER-compatible tracks, such as avoiding music with a strong beat, but the SSM group’s choices were not constrained in this way and reflected a mix of classical and rock genres. Personal preferences may also have played a limited role. Upon analyzing the qualities of the SSM group (Multimedia Appendix 2), 2 participants selected tracks that would qualify as having elements overlapping with CER, while the other 3 were characterized by complexity, dynamic tempos and textures, and/or heavy beats.

Participants in the SSM group showed a marked improvement in HRV, a result that was not observed in the PM or control groups. This finding suggests that familiar music, which aligns with individual preferences, may offer unique benefits. The intrinsic qualities of the music—how they interact with individual taste and exposure—remain an important area for further investigation.

While the SSM group exhibited modest improvements in sleep, including longer time in bed and increased sleep duration, no significant changes in sleep latency, REM or SWS sleep, or sleep efficiency were observed in either the PM or SSM groups. These findings may reflect the erratic sleep patterns of surgeons, with their on-call schedules and other work-related factors influencing sleep outcomes. Despite the promising results observed for FC and HRV, the lack of significant changes in sleep parameters highlights the complexity of the relationship between music intervention and sleep quality. It suggests that further refinement of intervention protocols may be necessary to achieve significant improvements in sleep, particularly in this high-stress, highly variable work environment. In particular, larger sample sizes and longer follow-up periods could prove more resilient to missing actigraphy data and confounding variables based on the inherent variability in surgeon sleep patterns and schedules, which may have influenced the sleep time and duration. Larger samples in the SSM group would also afford a broader range of SSM to explore the compositional elements of what participants select as their perceived (versus physiologically) relaxing music playlist.

### Strengths and Limitations

This study design has several strengths. First, the study focused on surgeons, a profession known to be accompanied by erratic sleep patterns and exposure to occupational noise. Second, the study design incorporated high-precision tools for outcomes assessment. Third, the study randomized participants into music intervention groups, controlling both for no music within the nighttime routine and the use of music that may not possess all the desired CERs for maximum therapeutic benefit, thus addressing longstanding mechanistic questions via an appropriately comparative method.

However, these strengths also had their drawbacks. One of the inherent challenges of conducting a real-world study is attributing observed effects specifically to the intervention, rather than to unrelated life events or external factors—especially with a small sample size. Despite these limitations, the changes in FC observed in both music groups stood out as noteworthy and consistent. Whereas targeting surgeons allowed investigators to assess the music intervention on both FC and sleep quality outcomes, few participants completed this study with data sufficient for analysis. Furthermore, due to the circumstantial imbalance in gender among our surgeons at the time and 2 female participants dropping out of the study, the majority were male, which weakened the overall generalizability of the results. Participant withdrawals were primarily due to unpredictable schedules and personal life events, highlighting a key challenge in conducting studies with high-demand populations. None of the surgeons reported dissatisfaction with the study design. Full-scale study recruitment targets should account for attrition and further refine recruitment strategies to ensure more balanced gender representation.

HRV, while an important marker of autonomic nervous system function and well-being [26,27], has limitations due to significant individual variances influenced by age, gender, and hydration, necessitating personalized analysis for accuracy. Sleep's relationship with HRV is complex; both excessive and insufficient sleep may not directly reflect recovery levels, as underlying causes such as illness or stress impact HRV. WHOOP, acknowledging these nuances, minimally incorporates sleep into HRV calculations, focusing on measurements during SWS for consistency, as HRV data from other sleep stages, particularly REM, are more variable and less reliable.

The use of high-precision tools for measuring outcomes provides confidence in the changes observed despite the small sample size. However, the specialized nature of the 7-Tesla fMRI limits its availability for broader population studies, making wearable actigraphy a useful alternative when fMRI is not feasible.

The music selection for the PM group only included examples from Western classical music, because the majority of prior evidence-based music intervention studies used this repertoire [56,57]. Meanwhile, the self-selected playlist was completely at the discretion of each participant and largely featured other genres. Given participant preferences, it would be worthwhile to examine selections from these other genres that incorporate the CERs. In addition, studies have shown that participant agency in selecting their listening tracks can positively impact music therapy [58]. It remains an open question to what degree it was the music itself or the participants’ self-reliance that produced the observed effects on HRV in the SSM group.

We selected ICU noise as the control stimulus during fMRI scanning. We acknowledge that ICU noise is itself a known stressor and therefore may elevate baseline arousal; however, we consider this a feature rather than a flaw in our design, because it frames PM and SSM as potential modulators of stress-related neural and physiologic responses under credible real-world auditory strain in our target population’s working environment.

There were a few other limitations. Participants used their personal smartphones to capture actigraphy data. Reminding participants to update the app on their smartphone was a mild deterrent. Compliance was self-reported; an automated system might be more reliable. Finally, the study took place during the COVID-19 pandemic, which added significant challenges to participant recruitment, scheduling, and data collection. These factors likely influenced the overall completion rate and may have introduced additional confounding variables. Additionally, as no standardized restrictions were imposed regarding hydration status, caffeine and alcohol intake, or physical activity during the 6-week monitoring period, individual variabilities in these factors may potentially influence HRV measurements and therefore represent potential sources of variability in the physiological data.

### Conclusion

This pilot study tested an innovative multimodal protocol for evaluating music as an intervention, incorporating neuroimaging, wearable actigraphy, and psychological questionnaires. Each of these research tools revealed distinct insights into the potential effects of music interventions. For example, fMRI findings suggest that music may reduce attentional reactivity to ICU noise and help preserve FC in regions linked to empathy, emotional regulation, and bodily awareness—effects not observed in the control group. These subtle neural shifts would likely have gone undetected using only traditional self-report or wearable data, highlighting the value of multimodal assessment in stress research. In contrast, SSM at bedtime was associated with increased HRV, an outcome not addressed by neuroimaging data.

Results regarding sleep were more equivocal, likely due to the small sample size and the unpredictable schedules of the surgeon participants. Taken together, this preliminary data suggests that music could serve as a nonpharmacological, cost-effective, and safe intervention for preserving empathic neural pathways and improving HRV—a key indicator of improved health.

As a pilot study, these findings provide valuable insights but must be interpreted with caution due to the study's small sample size and feasibility design. Larger and longer-term studies are warranted to further evaluate the potential of music interventions in reducing stress and burnout and improving well-being for those working in high-stress occupations, with this study providing a promising, multimodal methodology that could be adapted for future research on the effects of music in stress management and cognitive well-being.

## Data Availability

The datasets generated or analyzed during this study are not publicly available due to the small and potentially reidentifiable number of participants and the nature of the mental health and imaging data; data are available from the corresponding author on reasonable request and pending an institutional review board–approved collaborative study if deemed necessary.

## Appendix

Multimedia Appendix 1 Brain network assignment of Talairach regions and coordinates.

Multimedia Appendix 2 Self-selected music repertoire.

Multimedia Appendix 3 CONSORT checklist for pilot feasibility studies.

## Abbreviations

AFNI: Analysis of Functional NeuroImages
CER: compositional elements of relaxation
FC: functional connectivity
fMRI: functional magnetic resonance imaging
GEE: generalized estimating equations
HR: heart rate
HRV: heart rate variability
ICU: intensive care unit
PI: principal investigator
PM: prescribed music
REM: rapid eye movement
SSM: self-selected music
SWS: slow-wave sleep
