# Supplementary material for: Impact of Prescribed and Self-Selected Music Interventions on Stress, Sleep, Heart Rate Variability, and Brain Connectivity in Surgeons Using 7-Tesla Functional Magnetic Resonance Imaging and Wearable Actigraphy: Multimodal Feasibility Randomized Controlled Trial
Source: JMIR Form Res. 2026 Apr 17;10:e84899. doi: 10.2196/84899 (PMC13135162; doi:10.2196/84899)
Supplement: Multimedia Appendix 1 [file formative_v10i1e84899_app1.docx]

**Brain Network Assignment of Talairach Regions and Coordinates**

The following brain regions were identified based on the Talairach TT_icbm452 atlas as reference included in the Analysis of Functional NeuroImages (AFNI) software package. Each region was matched to one or more functional brain networks using anatomical name patterns. The assignments were based on established literature and used to summarize group-level brain network activity. Shell scripts together with R were used to extract connectivity values, compute statistics, and associate regions to networks. Some brain regions were associated with multiple networks, reflecting their overlapping roles in different functional domains. The table below lists all region-to-network assignments used in the manuscript’s analysis (Table S1) with additional details on precise coordinates (Table S2).

**Table S1**. Network assignments mapped to Talairach brain regions

| **Network** | **Brain region** |
| --- | --- |
| Default | Angular gyrus |
|  | Posterior cingulate |
|  | Precuneus |
| Dorsal attention | Superior parietal lobule |
| Frontoparietal | Inferior frontal gyrus |
|  | Middle frontal gyrus |
| Limbic | Amygdala |
|  | Anterior cingulate |
|  | Cingulate gyrus |
|  | Hippocampus |
|  | Parahippocampal gyrus |
|  | Posterior cingulate |
| Somatomotor | Paracentral lobule |
|  | Postcentral gyrus |
|  | Precentral gyrus |
| Stress | Amygdala |
|  | Anterior cingulate |
|  | Hippocampus |
|  | Insula |
| Ventral attention | Inferior parietal lobule |
|  | Insula |
|  | Supramarginal gyrus |

**Table S2**. Coordinates for brain regions.

| **Brain regions** | **Coordinates** |
| --- | --- |
| Left hippocampus | "29.1667 23.3333 -8.75" |
| Left amygdala | "21.5 4.5 -16" |
| Left posterior cingulate | "8.77273 52.3182 13.9545" |
| Left anterior cingulate | "7.05696 -32.8165 8.51266" |
| Left subcallosal gyrus | "9.72222 -11.6667 -12.5" |
| Left transverse temporal gyrus | "47.7941 21.6177 12.5" |
| Left uncus | "25.2027 1.41892 -28.0405" |
| Left rectal gyrus | "5 -25 -22.5" |
| Left fusiform gyrus | "37.0055 55.3022 -14.0385" |
| Left inferior occipital gyrus | "35.1562 87.0312 -6.71875" |
| Left inferior temporal gyrus | "51.625 26.125 -20.375" |
| Left insula | "37.9167 6.52778 9.07407" |
| Left parahippocampal gyrus | "24.5952 22.6905 -12.7857" |
| Left lingual gyrus | "12.1907 76.5206 -2.96392" |
| Left middle occipital gyrus | "35.3455 80.2236 5.63008" |
| Left orbital gyrus | "7.88461 -43.2692 -25.5769" |
| Left middle temporal gyrus | "51.0171 38.6787 -0.579849" |
| Left superior temporal gyrus | "50.3866 14.7337 -0.798969" |
| Left superior occipital gyrus | "35.7143 80.7143 26.7857" |
| Left inferior frontal gyrus | "42.5784 -23.3627 1.89216" |
| Left cuneus | "12.2929 83.0917 17.145" |
| Left angular gyrus | "42.8333 66.8333 32.5" |
| Left supramarginal gyrus | "50.2273 47.9546 29.2046" |
| Left cingulate gyrus | "8.36826 11.0928 33.1287" |
| Left inferior parietal lobule | "46.6212 39.6515 36.2273" |
| Left precuneus | "12.4231 60.0641 38.8077" |
| Left superior parietal lobule | "25.4592 59.1327 49.949" |
| Left middle frontal gyrus | "36.0219 -29.3252 24.4023" |
| Left paracentral lobule | "6.42857 31.1905 51.6667" |
| Left postcentral gyrus | "40.6507 25.7877 43.4247" |
| Left precentral gyrus | "43.2583 7.80806 36.0782" |
| Left superior frontal gyrus | "17.1505 -40.6459 25.6003" |
| Left medial frontal gyrus | "7.72727 -27.7955 22.0682" |
| Left lentiform nucleus | "21.4167 1.08333 2.58333" |
| Left hypothalamus | "5 2.5 -5" |
| Left red nucleus | "5 17.5 -2.5" |
| Left substantia nigra | "9.16667 15.8333 -5.83334" |
| Left claustrum | "30.2778 -2.5 5.27778" |
| Left thalamus | "11.6525 18.4322 7.16102" |
| Left caudate | "11.3333 -6.5 8.66667" |
| Left caudate tail | "25.8333 32.5 9.16666" |
| Left caudate body | "10.8333 -6.54762 13.4524" |
| Left caudate head | "9.5 -14.5 0.5" |
| Left ventral anterior nucleus | "10.8333 4.16667 10.8333" |
| Left ventral posterior medial nucleus | "12.5 17.5 7.5" |
| Left ventral posterior lateral nucleus | "17.5 19.1667 4.16666" |
| Left medial dorsal nucleus | "5.35714 14.6429 8.21429" |
| Left lateral dorsal nucleus | "0 15 10" |
| Left pulvinar | "15.3947 26.1842 7.76315" |
| Left lateral posterior nucleus | "17.5 17.5 15" |
| Left ventral lateral nucleus | "12.5 10.8333 7.5" |
| Left midline nucleus | "7.5 17.5 17.5" |
| Left anterior nucleus | "7.5 10 15" |
| Left mammillary body | "12.5 17.5 2.5" |
| Left medial globus pallidus | "14.1667 4.16667 -0.833336" |
| Left lateral globus pallidus | "17.0833 2.08334 0.416664" |
| Left putamen | "22.9255 0.159576 3.45744" |
| Left nucleus accumbens | "12.5 -12.5 -7.5" |
| Left medial geniculum body | "17.5 22.5 -2.5" |
| Left lateral geniculum body | "22.5 22.5 -2.5" |
| Left subthalamic nucleus | "10 12.5 -2.5" |
| Left cerebellar tonsil | "2.5 62.5 -32.5" |
| Left inferior semi-lunar lobule | "2.5 72.5 -27.5" |
| Left fastigium | "0 15 10" |
| Left nodule | "2.5 72.5 -17.5" |
| Left uvula | "2.5 62.5 -5" |
| Left pyramis | "28.1015 50.6579 -37.2744" |
| Left culmen | "27.943 70.1582 -39.5886" |
| Left declive | "7.5 52.5 -22.5" |
| Left dentate | "6.875 53.125 -27.5" |
| Left tuber | "8.88889 69.4444 -30.5556" |
| Left cerebellar lingual | "30.2885 73.6539 -30.7692" |
| Right hippocampus | "18.5902 45.6955 -15.9962" |
| Right amygdala | "24.1434 69.1434 -18.6538" |
| Right posterior cingulate | "14.1667 54.7222 -22.5" |
| Right anterior cingulate | "42.8571 71.5179 -26.6964" |
| Right subcallosal gyrus | "5 45 -12.5" |
| Right transverse temporal gyrus | "-0.625 -29.375 -26.6667" |
| Right uncus | "-0.340912 -23.5227 -9.65909" |
| Right rectal gyrus | "-30.3571 23.9286 -8.21429" |
| Right fusiform gyrus | "-23.6538 4.80769 -15.5769" |
| Right inferior occipital gyrus | "-10 52.6667 13.75" |
| Right inferior temporal gyrus | "-8.71053 -32.7632 7.3421" |
| Right insula | "-11.3095 -12.0238 -12.5" |
| Right parahippocampal gyrus | "-52.2222 20.2778 12.5" |
| Right lingual gyrus | "-26.8421 2.63158 -28.6842" |
| Right middle occipital gyrus | "-8.26923 -24.8077 -22.5" |
| Right orbital gyrus | "-39.1848 54.5109 -13.8587" |
| Right middle temporal gyrus | "-35.9483 87.3276 -7.5" |
| Right superior temporal gyrus | "-52.9706 25.9706 -20.5588" |
| Right superior occipital gyrus | "-40.8486 5.61927 8.96789" |
| Right inferior frontal gyrus | "-25.9158 22.0049 -12.5495" |
| Right cuneus | "-14.4307 77.0049 -2.84653" |
| Right angular gyrus | "-37.8571 80.2381 5.79366" |
| Right supramarginal gyrus | "-7.83334 -42.8333 -25.1667" |
| Right cingulate gyrus | "-53.2117 39.1241 -0.36496" |
| Right inferior parietal lobule | "-51.9982 15.3495 -0.779572" |
| Right precuneus | "-37.7941 80.4412 27.2059" |
| Right superior parietal lobule | "-45.56 -23.78 2.25999" |
| Right middle frontal gyrus | "-14.3314 83.4593 16.9186" |
| Right paracentral lobule | "-45.8333 66.1667 32.5" |
| Right postcentral gyrus | "-52.9762 48.5714 29.5238" |
| Right precentral gyrus | "-9.59596 9.92424 32.6515" |
| Right superior frontal gyrus | "-49.7353 39.6176 35.7353" |
| Right medial frontal gyrus | "-13.4908 59.9885 38.7212" |
| Right lentiform nucleus | "-28.5638 59.0957 50.1596" |
| Right hypothalamus | "-37.8974 -29.3846 24.8077" |
| Right red nucleus | "-7.28723 30.1596 50.9043" |
| Right substantia nigra | "-44.0839 25.2329 42.7485" |
| Right claustrum | "-45.4524 8.0238 36.3333" |
| Right thalamus | "-19.2012 -40.4438 25.8136" |
| Right caudate | "-9.15179 -26.8527 22.1652" |
| Right caudate tail | "-22.9237 0.296616 2.33051" |
| Right caudate body | "0 15 10" |
| Right caudate head | "-5 17.5 -2.5" |
| Right ventral anterior nucleus | "-10.8333 17.5 -7.5" |
| Right ventral posterior medial nucleus | "-32.8571 0.714287 6.42857" |
| Right ventral posterior lateral nucleus | "-13.0932 17.9237 7.33051" |
| Right medial dorsal nucleus | "-11.5625 -9.0625 8.125" |
| Right lateral dorsal nucleus | "-20 27.5 17.5" |
| Right pulvinar | "-12.2619 -7.2619 13.2143" |
| Right lateral posterior nucleus | "-9.58334 -13.3333 0.416664" |
| Right ventral lateral nucleus | "-12.5 5 12.5" |
| Right midline nucleus | "0 15 10" |
| Right anterior nucleus | "-17.5 17.5 5" |
| Right mammillary body | "-6.07143 14.6429 7.5" |
| Right medial globus pallidus | "-12.5 17.5 17.5" |
| Right lateral globus pallidus | "-16.1842 26.9737 6.97369" |
| Right putamen | "-17.5 17.5 15" |
| Right nucleus accumbens | "-14.6429 11.7857 9.64286" |
| Right medial geniculum body | "-7.5 17.5 17.5" |
| Right lateral geniculum body | "-7.5 4.16667 10.8333" |
| Right subthalamic nucleus | "-9.16666 15.8333 -0.833336" |
| Right cerebellar tonsil | "-15.625 3.75 -0.625" |
| Right inferior semi-lunar lobule | "-22.0455 7.04545 0.227276" |
| Right fastigium | "-24.0556 -2.16666 3.27778" |
| Right nodule | "-12.5 -10 -7.5" |
| Right uvula | "-17.5 22.5 -2.5" |
| Right pyramis | "-22.5 22.5 -2.5" |
| Right culmen | "0 15 10" |
| Right declive | "-2.5 65 -32.5" |
| Right dentate | "-2.5 75 -27.5" |
| Right tuber | "0 15 10" |
| Right cerebellar lingual | "-2.5 71.25 -18.75" |
