# Supplementary material for: Impact of Prescribed and Self-Selected Music Interventions on Stress, Sleep, Heart Rate Variability, and Brain Connectivity in Surgeons Using 7-Tesla Functional Magnetic Resonance Imaging and Wearable Actigraphy: Multimodal Feasibility Randomized Controlled Trial
Source: JMIR Form Res. 2026 Apr 17;10:e84899. doi: 10.2196/84899 (PMC13135162; doi:10.2196/84899)
Supplement: Multimedia Appendix 2 [file formative_v10i1e84899_app2.docx]

**Appendix 2. Self-selected music repertoire**. Each participant randomized to the self-selected music group was instructed to select their preferred music that they find to promote relaxation.

| Title | Composer/Artist | Description | Tempo |  |
| --- | --- | --- | --- | --- |
| Participant 002 | | | | |
| Bohemian Rhapsody | Queen | Blend of rock and opera with ballad and up-tempo sections; lyrics in English | Ballad ca. ♩ = 68 bpm  Up-tempo ca. ♩ = 124 bpm |  |
| Participant 006 | | | | |
| Trois Gymnopédies | Erik Satie |  |  |  |
| Gymnopédie No. 1 |  | Instrumental piano, melancholic, instructed to be played “lent et douloureux,” meaning *slowly with pain/grief* | Ca. ♩ = 72-79 bpm |  |
| Gymnopédie No. 2 |  | Instrumental piano, dreamlike, introspective, instructed to be played “lent et triste,” meaning *slow and sad* | Ca. ♩ = 66-68 bpm |  |
| Gymnopédie No. 3 |  | Instrumental piano, dreamlike, introspective, instructed to be played “lent et triste,” meaning *slow and sad* | Ca. ♩ = 99 bpm |  |
| Participant 012 | | | | |
| Symphony No. 9 in D minor, Op. 125 | Ludwig van Beethoven |  |  |  |
| Movement I |  | Orchestral, classical, instructed to be played “allegro ma non troppo, un poco maestoso,” meaning *quickly but not too fast, a little majestic* | ♩ = 88 bpm |  |
| Movement II |  | Orchestral, classical, with variable segments, instructed to be played “Molto vivace,” meaning *very lively* and “Presto” meaning *fast* | 𝅗𝅥𝅭 = 116 bpm  𝅝 = 116 bpm |  |
| Movement III |  | Orchestral, classical, with variable segments, instructed to be played “Adagio molto e cantabile,” meaning *very slow and in a singing style* and “Andante moderato” meaning *moderately slow* | ♩ = 60 bpm  ♩ = 63 bpm |  |
| Movement IV |  | Orchestral, classical, with variable segments, generally quick tempo and majestic | Variable, ranging from  ♩ = 60 bpm to  𝅝 = 132 bpm |  |
| Participant 013 | | | | |
| Sur L’Ocean Couler de Fer | Alcest | Alternative, Guitar and voice, lyrics in French | ♩ = 67 bpm |  |
| Participant 019 | | | |  |
| Aqualung | Morcheeba | Trip hop, electronic, heavy beat, lyrics in English | ♩ = 93 bpm |  |
